# Syncytium: the viral escape room secret to persistent infection of SARS-CoV-2

**DOI:** 10.3389/fmicb.2025.1561274

**Published:** 2025-06-04

**Authors:** Oksana Palchevska, Francisco Dominguez

**Affiliations:** ^1^Department of Microbiology, School of Medicine, University of Alabama at Birmingham, Birmingham, AL, United States; ^2^Regional Biocontainment BSL-3 Research Laboratory (SEBLAB), University of Alabama at Birmingham, Birmingham, AL, United States

**Keywords:** persistent infection, SARS-CoV-2, syncytia, cell-cell fusion, long COVID

The coronavirus disease (COVID-19) pandemic has unveiled a complex spectrum of disease manifestations, from acute respiratory distress to persistent health effects. In its acute phase, COVID-19 unleashes a storm of lung injury, marked by diffuse alveolar damage, thrombosis, and inflammation (Bussani et al., 2020). Yet, for many, the battle doesn't end with recovery from acute symptoms. A significant number of patients experience prolonged health issues known as “long COVID-19” (Davis et al., 2023) or post-acute sequelae of SARS-CoV-2 (PASC; Swank et al., 2023), challenging our understanding of viral infections and recovery. This condition is also referred to as “persistent SARS-CoV-2 infection,” where the virus persists beyond 2 months post-initial onset (Furie et al., 2023). Another phenomenon, termed “walking pneumonia,” encompasses a range of persistent viral respiratory infections symptoms, such as those of SARS-CoV-2 infection, rather than a specific diagnosis (Walking Pneumonia: What You Should Know, n.d.). The burning question remains: does this persistence represent an extended infectious cycle, or does the virus truly transitions into a persistent state? We propose the hypothesis that SARS-CoV-2 transitions to persistent infection, facilitated by syncytia formation, which may be key to unlocking the unknowns of long COVID-19.

**Figure 1:**
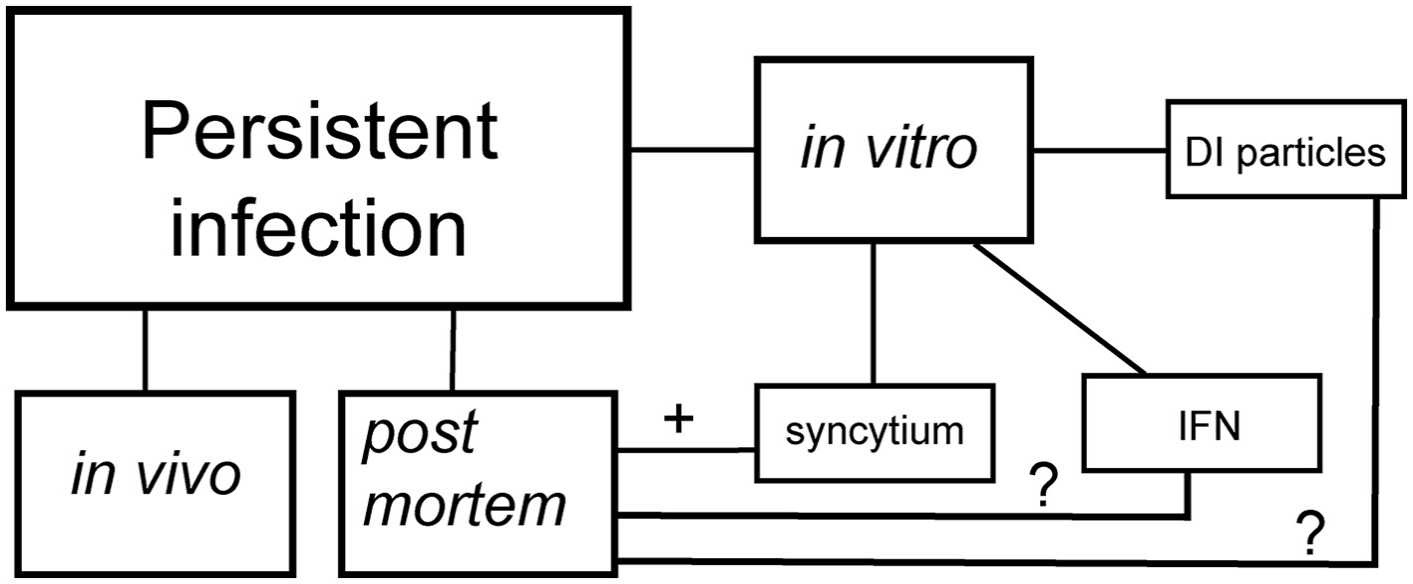
Schematic presentation of persistent SARS-CoV-2 infection. DI particles—the establishment of persistent infection via the emergence of defective interfering particles. IFN—the establishment of persistent infection via the interferon selection. Syncytium—the establishment of persistent infection via formation of syncytia.

RNA viruses (i.e., alphaviruses) employ sophisticated strategies to avoid host defenses and develop persistent infection. Two fascinating mechanisms have emerged for RNA viruses (Schlesinger and Schlesinger, 1986)—both begin with a cytopathic viral assault, but their paths later diverge (Figure 1). In one path, the virus transitions to formation of defective interfering (DI) particles, a defective viral genome that is capable of replication and propagation in the presence of helper genome or wild type virus (Girgis et al., 2022; Weaver et al., 1999). The alternative strategy occurs when the virus overcomes the interferon defense, leaving only a small fraction of infected cells as survivors (Schlesinger and Schlesinger, 1986). These observations come from *in vitro* experiments, have now been confirmed in the more complex realm of coronaviruses. Syncytium, sometimes also called a “giant cell,” is a large, multi-nucleated cell that is born from cell-cell fusions (Zimmerberg et al., 1993). While syncytia naturally occur in some tissues, such as muscles, as a part of normal growth (Zimmerberg et al., 1993), their appearance can also be a sign of a more pathological outcome (Rajah et al., 2022). This cellular fusion is orchestrated by an ensemble of transmembrane and membrane-associated proteins of diverse origin (Ashorn et al., 1993; Mohan, 1992). Apart from DI particles, syncytia also serve as another hallmark of SARS-CoV-2′s persistence. Syncytia formation is a process that SARS-CoV-2 has mastered with outstanding efficiency. *In vitro* research on SARS-CoV-2 evolution has revealed that the spike protein that contains furin cleavage site (FCS) drives virus toward utilization of Angiotensin-Converting Enzyme 2 (ACE2)/Transmembrane serine protease 2 (TMPRSS2) receptors and syncytia formation (Frolova et al., 2022; Shiliaev et al., 2021). In the case of viral infection-induced syncytia formation, it requires two key players: cellular receptors/co-receptors and viral spike proteins (Yin et al., 2024; Zimmerberg et al., 1993). Together, they transform the cellular landscape into a playground for viral persistence.

The virus-induced syncytia formation phenomenon has been observed across a diverse phylogenetic spectrum, from influenzas to HIV-1 and HIV-2, from the highly contagious measles to respiratory syncytial virus (Zimmerberg et al., 1993). The SARS and MERS coronaviruses, closely related to SARS-CoV-2, have also demonstrated this cellular merger in infected tissues (Bussani et al., 2020; Chan et al., 2013; Franks et al., 2003; Hoffmann et al., 2020; Matsuyama et al., 2010; Qian et al., 2013; Tian et al., 2020), proving that syncytia formation is not merely a laboratory curiosity observed *in vitro* (Buchrieser et al., 2020). For viruses, this cellular fusion ability confers an outstanding advantage, helping them evade antibody neutralization and avoid the broader immune response (Frolova et al., 2022; Rajah et al., 2022). Moreover, as demonstrated in HIV-1 and HIV-2, it can serve as an alternative highway for infection spread (Pearce-Pratt and Phillips, 1993).

In the intricate dance of viral infection, TMPRSS2 emerges as a key choreographer. While not essential for host cell fusion *in vitro*, its presence significantly enhances the process (Frolova et al., 2022; Shiliaev et al., 2021). This leads us to an intriguing hypothesis: could TMPRSS2 expression in the population be a limiting factor for syncytia formation and, by extension, SARS-CoV-2 persistence *in vivo*? This could shed light on the puzzling presence of SARS-CoV-2 N protein in some endothelial cells of *postmortem* human lungs (Swank et al., 2023). Furthermore, several groups have demonstrated that the syncytia formed during SARS-CoV-2 infection are formidable, as they are resistant to both humoral immunity and most anti-COVID drugs. These fused cells may also act as viral factories, producing S protein that sheds, fueling the inflammatory fire (Frolova et al., 2022; Swank et al., 2023). Yet, the question of whether these viruses can use syncytia as intercellular bridges for spread *in vivo* remains a mystery.

In the ongoing saga of long COVID-19, one compelling theory points to persistent viral reservoirs lurking in certain tissues. These viral hideouts could nestle in neuronal tissue, brain glia, the vagus nerve, endothelium, or bronchi, potentially arising from survival selection under interferon pressure.

Autopsy of *postmortem* lung tissue from confirmed SARS-CoV-2 cases has revealed a landscape dotted with abnormal pneumocytes and syncytia, both pseudo and genuine (Bussani et al., 2020). These findings are invaluable, offering a frozen snapshot of syncytia formation *in vivo*, almost impossible to observe in real-time in living subjects. Intriguingly, affected tissues are often those less accessible to immune cells or hidden by barriers in the central nervous system (CNS; Daneman and Prat, 2015; Gopallawa et al., 2023).

In a twist of viral persistence, some COVID-19 cases have shown a delayed viral presence, with particles detected beyond 3 weeks post-infection, coinciding with significant lung fibrosis (Bussani et al., 2020; Swank et al., 2023). The authors suggested the hypothesis of a remote viral influence, where the pathogen's effects linger long after its clearance, possibly through the action of shed S1 protein (Frolova et al., 2022).

Unlike its syncytia-forming viral cousins such as HIV or CMV, SARS-CoV-2 hasn't yet been caught causing latent or persistent infection, meaning it cannot integrate its genetic material into host cell genomes. However, as a member of the RNA+ group of viruses, SARS-CoV-2 might have a few tricks for establishing persistence. Three potential strategies emerge: the development of DI particles, the selection of specific mutations, or the modulation of interferon production (Weiss et al., 1980). While interferon modulation seems to align best with observations from acute COVID-19 autopsies (Swank et al., 2023), we can't rule out the DI particle strategy. These viral particles have been spotted *in vitro* (Girgis et al., 2022), tempting us to wonder if they might also be playing a similar, low frequency game *in vivo*. Reports of SARS-CoV-2 lingering in pneumocytes and endothelial cells (Bussani et al., 2020) have been attributed to slow viral replication, but could syncytia-mediated spread be buying the virus extra time to evade clearance?

In conclusion, we find ourselves facing a gap in our understanding of how SARS-CoV-2 outmaneuvers the immune response to establish persistent infection. The possibility that syncytia serve as viral “escape rooms” requires deeper investigation. Additionally, the potential role of DI particles *in vivo* remains an open question, calling for further research to unravel these viral mysteries.
